# Economic precarity shapes engagement in sex work and HIV-related behaviors among African refugee male sex workers in Italy: A mixed-methods study

**DOI:** 10.21203/rs.3.rs-8289248/v1

**Published:** 2026-02-11

**Authors:** Marco Barracchia, Samira Shirzaei Nichols, Shamrock Osman Wumpini, Gamji Rabiu Abu-Ba’are

**Affiliations:** Maastricht University; University of Central Arkansas; University of Rochester; University of Rochester

**Keywords:** African refugees, male sex workers, economic precarity, HIV vulnerability, mixed-methods, Italy

## Abstract

**Background:**

African refugee male sex workers (ARMSWs) in Europe experience overlapping forms of marginalization, including racism, legal precarity, and exclusion from formal labour markets, which collectively shape HIV vulnerability. However, little is known about how economic precarity drives engagement in sex work and HIV-related behaviors among ARMSWs in Italy.

**Methods:**

We used a sequential exploratory mixed-methods design within the Refugee Initiative for Sexual Health (RIfESH). First, we conducted 20 in-depth interviews and 2 focus group discussions with ARMSWs in Verona, Turin, and Milan to explore pathways into sex work, migration-stage experiences, and health-care access. Qualitative themes informed the development of a structured REDCap survey administered to 150 ARMSWs recruited through venue-based and snowball sampling. We used descriptive statistics and chi-square tests with Cramér’s V to examine associations between economic drivers (e.g., need for food, rent, and lack of other income) and sexual risk-taking, HIV service awareness, health-care discrimination, and reliance on traditional medicine. Integration occurred through side-by-side comparison and a joint display.

**Results:**

Most participants (79%) relied on sex work as their sole income source, and 72% had entered sex work by age 25. Economic pressures related to rent, food, and having no other income were significantly associated with condomless sex, lower HIV service awareness, greater use of traditional medicine, and higher reports of hospital stigma and unmet health-care needs. Qualitative narratives showed how poverty, migration-stage coercion, racism, and client power constrained condom negotiation and access to care, often forcing participants to accept unsafe practices to survive.

**Conclusions:**

Engagement in sex work among ARMSWs in Italy is primarily a survival response to structural exclusion rather than individual “risk-taking.” HIV prevention and care must be coupled with economic empowerment, anti-racist and stigma-free health services, and policies that recognize male refugee sex workers as a priority population.

## Introduction

HIV remains a critical public health challenge across Europe, particularly among populations situated at the intersection of economic precarity, migration-related trauma, and sexual marginalization. Among these, African refugee male sex workers (ARMSWs) represent a group whose health vulnerabilities are shaped by overlapping structural, social, and economic determinants rather than individual behavior. Despite biomedical advances in HIV prevention, recent surveillance data indicate that the European region has not achieved sustained progress toward elimination targets. The European Centre for Disease Prevention and Control (ECDC) and the World Health Organization (WHO) Regional Office for Europe reported 112,883 new HIV diagnoses in 2023, including approximately 24,731 cases within EU/EEA countries, indicating that progress toward epidemic control has stalled (ECDC, 2024).

In Italy, where migration from Sub-Saharan Africa remains a defining social reality, HIV incidence has shown a concerning upward trend. The *Istituto Superiore di Sanit* (ISS) documented 2,349 new HIV acquisitions in 2023, reflecting a 24 percent increase compared with 2022 (Istituto Superiore di Sanit (ISS, 2023; Marvelli, 2025). Nearly one-third of these new acquisitions occurred among foreign-born individuals, and close to one in five among African men who have sex with men (MSM) (Istituto Superiore di Sanit (ISS, 2023). Although ISS surveillance reports that foreign-born individuals accounted for 36.9% of new HIV diagnoses in 2023, cases are classified only as “foreigners,” obscuring differences by region of origin, gender, and socioeconomic position that are essential for understanding vulnerability and targeting prevention efforts. Current HIV data in Italy underscore persistent inequities in prevention and care access, particularly for migrants who are excluded from formal employment, constrained by precarious legal status, and subject to layered stigma in both health systems and communities (Callahan & Loscocco, 2023). The intersection of migration, sexuality, and economic marginalization thus defines a syndemic context in which HIV risk is structurally produced rather than individually chosen.

### Structural and Contextual Drivers of HIV Vulnerability

European research increasingly situates HIV vulnerability among migrants within broader systems of institutional violence, labor exclusion, and racialized stigma (Platt et al., 2013; Probst, 2023). Quantitative surveillance highlights elevated rates of late diagnosis among migrants, while qualitative studies illuminate how restricted employment opportunities, unstable housing, and discrimination limit agency in health-seeking behavior (Callahan & Loscocco, 2023; Mai, 2018). In Italy, these dynamics are compounded by restrictive migration policies and an under-resourced public-health infrastructure, which together constrain access to HIV testing, pre-exposure prophylaxis (PrEP), and culturally competent care.

Ethnographic evidence across Europe underscores that migrant sex workers’ exclusion from health-insurance systems often reflects moralized distinctions between “deserving” and “undeserving” citizens, reinforcing structural vulnerability (Probst, 2023). Similar patterns appear along migration routes: ARMSWs traveling through Niger, Algeria, and Libya often rely on transactional sex as a survival strategy amid violence, exploitation, and statelessness. The United Nations High Commissioner for Refugees (UNHCR, 2020) and other humanitarian reports document widespread sexual coercion and abuse along the Central Mediterranean corridor, illustrating how vulnerability precedes arrival in Europe. Upon reaching Italy, many refugees confront unemployment and housing insecurity that perpetuate dependence on sex work for survival (Abu-Ba’are et al., 2025; Mai, 2018). Racial and ethnic health inequities further compound these structural barriers and are central to understanding HIV vulnerability among African migrants in Europe. African-born populations experience disproportionately higher rates of HIV acquisition, late diagnosis, and reduced access to prevention services compared with Europeans, patterns rooted in systemic anti-Black racism, xenophobia, and discriminatory clinical practices (Logie et al., 2011; Mai, 2018; Platt et al., 2013; Probst, 2023; Shamrock et al., 2023). Migrants from Sub-Saharan Africa routinely report racialized stigma, biased treatment, and moralizing attitudes in clinical encounters, which discourage testing and continuity of care. Beyond healthcare, racial profiling, exclusion from formal labor markets, and housing discrimination deepen the socioeconomic precarity that ultimately shapes sexual negotiation, client dependence, and access to HIV-prevention tools. For ARMSWs, these racialized inequities intersect with legal precarity and sexual stigma, producing compounded vulnerabilities that heighten HIV exposure throughout migration and settlement in Italy.

While global and regional HIV programs have increasingly targeted female and transgender sex workers, male refugee sex workers remain largely invisible in both research and policy frameworks. Studies across Europe show that food insecurity, housing instability, and lack of income correlate strongly with condomless sex and lower uptake of HIV testing or treatment (ECDC, 2024; The Lancet, 2023). Yet few have explored how economic coercion and client exploitation shape sexual health decision-making and health behaviors among African men engaged in sex work in Italy.

### Gaps in Existing Evidence

Despite advances in understanding migrant health, the literature remains fragmented. Quantitative surveillance reveals epidemiological trends but cannot explain the lived experiences driving them. Conversely, qualitative ethnographies expose mechanisms of marginalization but often lack empirical scope or generalizability. To date, no study has employed an integrated mixed-methods design to examine how economic drivers shape entry into sex work and HIV vulnerability among ARMSWs in Italy. This integration gap matters because HIV vulnerability in this population is not merely a product of individual risk-taking but reflects institutional inequities in employment, housing, and health-care access (Platt et al., 2013; Probst, 2023). Moreover, while Italy’s current HIV-prevention strategy emphasizes vulnerable groups, male sex workers are not explicitly recognized within national or regional prevention frameworks. The absence of disaggregated surveillance by gender identity and migration status continues to obscure this population’s needs (Istituto Superiore di Sanit (ISS, 2023). This invisibility sustains a gap between epidemiological recognition and programmatic response.

### Study Aim and Contribution

This study addresses this gap through a mixed-methods sequential exploratory design that investigates the economic and personal reasons for engaging in sex work among ARMSWs in Italy and examines how these drivers intersect with HIV vulnerability, health-care access, and stigma. The qualitative component explores participants’ lived experiences, revealing how coercion, poverty, and discrimination shape engagement in sex work from their home countries through migration and resettlement. The quantitative component measures the prevalence of key economic motivators (e.g., food, rent, lack of income) and their statistical associations with HIV risk behaviors, service awareness, and health-care discrimination. Integrating both strands allows for a deeper understanding of how structural forces manifest in individual health outcomes.

### Urgency and Policy Relevance

The timing of this study is particularly critical. The ECDC 2025 progress report shows that the EU/EEA remains off-track to meet Sustainable Development Goal 3.3, which aims to end the AIDS epidemic by 2030 (ECDC, 2025). Twenty-one of forty-seven European countries reported increases in new HIV diagnoses in 2023, reversing previous downward trends (ECDC, 2024). In Italy, the steady rise in new acquisitions and persistently high rates of late diagnosis reveal enduring gaps in prevention and care (Istituto Superiore di Sanit (ISS, 2023).

Simultaneously, migration flows from Sub-Saharan Africa continue, driven by economic instability and climate crises. The *International Organization for Migration* (IOM, 2024) and Shamrock et al. (UNHCR, 2023) estimate that thousands of African men reach Italy annually through irregular routes, many facing unemployment rates exceeding 60 percent and severe housing insecurity. For many newly arrived ARMSWs, sex work becomes the only viable livelihood strategy within conditions of unemployment, housing insecurity, and legal precarity, patterns consistently documented in recent ethnographic and humanitarian reports (Platt et al., 2013; Probst, 2023; UNHCR, 2020) and echoed in the present study’s qualitative findings.

By integrating quantitative and qualitative evidence, this study moves beyond descriptive epidemiology to offer structurally grounded explanations of HIV vulnerability. It contributes to ongoing European and global debates on migrant health by foregrounding the interplay between economic precarity, racism, and health-care stigma. Importantly, the findings provide a foundation for developing anti-racist, stigma-free, and economically empowering interventions tailored to the realities of ARMSWs, a population too often excluded from both academic research and public-health policy.

### Conceptual Framework

To interpret these intersecting dynamics and guide data integration, this study draws on two complementary frameworks: the Structural Vulnerability Framework and the Socioecological Model, which together illuminate how macro-level social structures and micro-level lived experiences shape HIV vulnerability among ARMSWs in Italy.

The Structural Vulnerability Framework conceptualizes health outcomes as products of intersecting social hierarchies, including class, race, gender, legal status, and labor conditions, that systematically constrain individual agency (Bourgois et al., 2017; Quesada et al., 2011). Within this framework, vulnerability arises not from personal risk behaviors but from exposure to structural forces such as criminalization, racism, and exclusion from employment and healthcare systems. For ARMSWs, these intersecting hierarchies manifest through restricted access to legal work, housing instability, and stigma in clinical settings, factors that collectively shape the conditions under which sex work is undertaken and health care is sought.

Complementing this lens, the Socioecological Model situates HIV vulnerability within multiple, interacting levels of influence: individual (knowledge, behaviors, trauma), interpersonal (client and peer relations), community (stigma, service access), and structural (policy, migration law, economic exclusion) (Bronfenbrenner, 1979; McLeroy et al., 1988). This multi-level view aligns with the mixed-methods design by capturing both quantitative patterns (e.g., unemployment, food insecurity, service utilization) and qualitative narratives (e.g., coercion, racism, stigma) across ecological layers. Integrating these frameworks allows this study to move beyond behavioral explanations of HIV risk toward a structural understanding of vulnerability, one that accounts for how legal, economic, and social marginalization converge to produce health inequities. This theoretical grounding also supports the study’s mixed-methods integration: quantitative findings reveal the prevalence and strength of structural drivers, while qualitative narratives explain their mechanisms and lived consequences. Together, these frameworks underscore the urgency of addressing not only individual behavior but also the structural conditions that shape life, work, and health among ARMSWs in Italy. These frameworks also inform how quantitative and qualitative strands are merged during interpretation.

## Methods

### Study Design

This study is part of the Refugee Initiative for Enhancing Sexual Health (RIfESH), a community–academic partnership between the Behavioral, Sexual, and Global Health (BSGH) Lab at the University of Rochester and Circolo Pink in Verona, Northern Italy focused on ARMSWs. We used a sequential exploratory mixed-methods design in which qualitative findings informed instrument development for the quantitative survey and were integrated at interpretation through a side-by-side synthesis. In the first phase, qualitative data from in-depth interviews and focus group discussions were analyzed to identify key themes such as economic hardship, migration-related trauma, and stigma shaping engagement in sex work. These insights guided the development of the quantitative survey, ensuring that variables and response options reflected participants’ lived realities. The quantitative phase then examined the prevalence and associations of the themes across a larger sample, allowing us to determine the extent to which qualitative findings were generalizable. Integration of both phases during interpretation enabled us to triangulate patterns, using qualitative material to explain statistical relationships and quantitative findings to confirm and contextualize qualitative narratives. This design is consistent with exploratory mixed methods approaches described in established methodological guidance and in previous RIfESH studies.

RIfESH operates through a community-engaged research model characterized by shared decision-making, mutual capacity-building, and iterative collaboration between academic researchers, peer researchers, and community partners. The approach emphasizes co-development of instruments, shared interpretation of data, and long-term partnership rather than extractive data collection. This study reflects those principles by involving Circolo Pink staff and ARMSWs peer researchers in every stage of the project, including instrument design, recruitment, data collection, and interpretive validation. Findings from this study are informing the design of future interventions and trials.

### Setting and Participants

This study was conducted in partnership with Circolo Pink (Pink Refugees), a community-based human services organization that has longstanding relationships with ARMSWs in Italy. Data collection took place in safe, accessible locations at Circolo Pink’s offices in Verona and a sister organization in Turin. Within the broader RIfESH partnership, mobility networks also extend to Milan, but the present study focused on participants in Verona and Turin. Circolo Pink has a long-standing presence across these cities, providing safe spaces, health referrals, and social support to African migrants engaged in sex work. Eligibility criteria required participants to be refugees from Sub-Saharan Africa, currently living in Italy, at least 18 years old, fluent in English or Italian, self-identifying as sex workers, and having engaged in sex work within the past six months. These criteria were applied identically in both qualitative and quantitative phases.

### Sampling and Data Collection

In the qualitative phase, we conducted 20 in-depth interviews and two focus group discussions with ARMSWs to explore motivations for entering sex work, migration experiences, and the socio-economic conditions shaping their engagement in sex work in both their home countries and Italy. Qualitative data collection followed the RIfESH framework, collected by two trained ARMSW peer-researchers and one anthropologist. The Principal Investigator conducted intensive training lasting one week that covered ethics, confidentiality, trauma-informed interviewing, safe referral procedures, and qualitative field documentation. Interviews and focus groups were conducted in English or Italian, with bilingual peers providing interpretation when necessary. All sessions were held in private rooms at Circolo Pink, were audio-recorded with consent, and were accompanied by detailed field notes. Transcripts were produced verbatim and de-identified. Thematic saturation was reached around the seventh to eighth interview at each site, but additional interviews were conducted to confirm the stability of emerging patterns. Participants in the qualitative phase were recruited through chain-referral and venue-based strategies. Focus group participants were recruited during their periodic meetings at Circolo Pink and were then invited to participate in in-depth interviews and to refer peers.

This reflects the hidden nature of the ARMSW population and the community-based structure of the partnership.

The quantitative phase involved administering a structured survey to 150 ARMSWs. The survey was developed directly from qualitative findings and programmed in REDCap, a secure mobile platform. Peer researchers distributed the survey via mobile devices and offered assistance when needed. Recruitment followed a combination of venue-based sampling during community meetings and snowball sampling through peer referrals. All survey data were collected anonymously and stored on encrypted servers maintained by the BSGH Lab. The quantitative phase relied on snowball sampling initiated by seeds from Turin, Milan, and Verona. These seeds recruited participants across their personal and work networks in Northern Italy, allowing recruitment to extend across multiple neighborhoods and informal sex-work venues.

### Interview Guide and Questionnaire Development

The semi-structured qualitative guide was co-developed by the BSGH Lab and Circolo Pink and covered economic and social drivers of sex work, migration stressors, interpersonal and institutional stigma, and access to HIV testing and other health services. The guide was pilot-tested with two participants, reviewed for cultural sensitivity by Circolo Pink staff, and revised collaboratively.

The quantitative questionnaire mirrored these domains. Items were drawn from validated measures where available and supplemented by locally developed items based on qualitative themes. This ensured that questions on economic drivers, healthcare stigma, reliance on traditional medicine, client coercion, and HIV-service awareness reflected the lived realities of ARMSWs.

### Training

Training was central to the RIfESH model. The PI trained peer researchers and Circolo Pink staff on human-subjects protection, trauma-informed interviewing, informed consent, secure data handling, REDCap procedures, and responding appropriately to emotional distress or disclosure of violence. The training emphasized co-learning and capacity exchange, positioning peer researchers as equal collaborators, managing the research and independently conducting data collection activities.

### Nature of Questions

The primary outcome of this study was empirical evidence on economic and personal reasons for engaging in sex work and how the economic reasons influence HIV exposure, explored through both qualitative and quantitative methods. For the qualitative component, a semi-structured interview guide was used to explore topics such as financial hardship, lack of employment opportunities, family responsibilities, precarity during migration, and forms of sexual exploitation. Sample questions included: “Why did you start doing sex work?”, “What were the economic or personal circumstances leading up to it?”, and “Have you ever felt forced or coerced into sex work?” For the quantitative survey, structured questions assessed participants’ socio-demographic background, economic motivations (e.g., rent, food, children), and experiences of unprotected transactional sex. The core outcome variables measured included whether sex work was the participant’s only income source (yes/no), motivations for entering sex work (for example, money for rent, food, or children, or having no other income option), whether the participant had ever engaged in unprotected sex due to financial pressure, and whether the participant had employment outside of sex work (yes/no). Additionally, non-core variables were included in the analysis to capture broader contextual factors. Socio-demographic characteristics assessed included age, gender identity, education level, marital status, number of children, religion, immigration status, and country of origin. Healthcare access and discrimination were evaluated through questions on experiences of stigma or discrimination in hospital settings, unmet healthcare needs, barriers to seeking care, and reliance on traditional or herbal medicine. HIV-related knowledge and behaviors were assessed by measuring awareness of HIV services and HIV self-testing, HIV testing history and results, general condom use behavior (not only under conditions of pressure), and knowledge of partner sexual history. Structural and psychosocial factors included experiences of client coercion or violence, experiences of racism and xenophobia, and lack of legal protection when abused or exploited. Each of these variables was measured using binary, categorical, or Likert-type responses. Although not central to the primary aim, these non-core variables were essential for understanding the broader socio-structural context shaping health vulnerabilities among ARMSWs and for exploring secondary associations with economic motivations. The demographic characteristics of the participants are shown in Table 1 of the results section.

### Data Management

All interviews and focus groups were audio-recorded with participant consent, transcribed verbatim by trained assistants, de-identified, and stored securely. REDCap survey data were collected anonymously. Both qualitative and quantitative data were stored on encrypted, password-protected BSGH servers with restricted access to ensure privacy, confidentiality, and data protection.

### Data Analysis

This analysis explores how economic motivations for entering sex work intersect with critical aspects of HIV vulnerability, healthcare access, and health-seeking behaviors. Economic drivers such as food insecurity, housing instability, and lack of alternative income sources are examined concerning individuals’ awareness of HIV services, reliance on traditional medicine, experiences of healthcare-related stigma, and sexual health practices. By identifying statistically significant associations across these domains, the findings shed light on how structural inequalities and financial hardship can shape health behaviors and risk exposure. Organizing the results thematically reveals how economic precarity compounds marginalization and influences health outcomes within this population.

### Qualitative Data Analysis

Analysis followed a summative content analysis approach (Hsieh & Shannon, 2005) incorporating interpretive thematic synthesis as described by Braun and Clarke (Braun & Clarke, 2006). The summative content analysis approach combines counting the frequency of key words or phrases with interpretive analysis of their contextual meaning. Each transcript was independently reviewed by two analysts who produced short analytic summaries highlighting salient economic and structural themes and patterns. These patterns were tabulated using Excel to record word frequencies and contextual notes. Coding, in this approach, functioned as an *intermediate step* to group related expressions rather than as an exhaustive codebook process. Analysts developed short analytic summaries (approximately 150–200 words per transcript) synthesizing recurrent ideas and illustrative quotations. Analysts then met to compare interpretations and reconcile differences. The PI reviewed all summaries and developed an analytic matrix that identified themes consistently appearing across transcripts, including economic hardship, migration trauma, healthcare discrimination, housing and food insecurity, and client coercion. The lead analyst revisited transcripts to verify interpretive accuracy and extract illustrative quotations. Circolo Pink staff reviewed emerging themes to confirm cultural and contextual accuracy. This procedure followed the summative content analysis approach and interpretive thematic synthesis procedures described in prior work involving AMRSWs. The analytic process included maintaining an audit trail of memos, discussions, and decision logs, contributing to transparency and rigor. This multi-step process emphasized triangulation, team validation, and transparency, ensuring analytic rigor while preserving participants’ lived contexts.

### Quantitative Data Analysis

Quantitative data were analyzed using descriptive statistics to summarize socio-demographic characteristics and economic indicators. We used chi-square tests of independence to assess associations between engaging in unprotected sex and economic motivators such as the need for food, rent, childcare, and lack of alternative income. These variables were selected because prior qualitative findings identified them as salient forms of economic pressure shaping sexual decision-making and because socioeconomic factors are well-recognized determinants of HIV vulnerability in the literature (Greener & Sarkar, 2010). Cramér’s V was used to assess the strength of association for each statistically significant relationship.

### Integration of Qualitative and Quantitative Phases

Integration occurred during two stages. First, qualitative themes directly informed the construction of quantitative survey items. Second, during interpretation, qualitative explanations were aligned with quantitative associations in a side-by-side synthesis. This process allowed statistical patterns, for example, the association between economic motivators and unprotected sex or health-service awareness, to be contextualized using narratives from the qualitative strand. This analytic approach is consistent with principles of mixed-methods integration described in previous RIfESH analyses.

### Trustworthiness and Reflexivity

To enhance trustworthiness, the team engaged in ongoing peer review and triangulation across IDIs and FGDs. The analytic process was documented through memo writing and discussion logs, creating an audit trail of analytic decisions. Summaries were shared with Circolo Pink staff for interpretive feedback and contextual validation, ensuring community perspectives informed final interpretations.

The research team’s reflexivity was central throughout the analysis. The qualitative analysts included two peer research assistants, both ARMSWs affiliated with Circolo Pink, whose lived experience informed the interpretation of stigma, discrimination, and healthcare exclusion. The principal investigator, an external researcher, maintained a reflexive stance through analytic journaling and regular consultation with community partners to mitigate outsider bias. This summative approach allowed the team to identify how frequently particular experiences were described while also interpreting their deeper social meanings, linking participants’ narratives to the syndemic framework guiding the study

The RIfESH mixed-method assessment is best classified as a community-engaged, sequential exploratory mixed-method study implemented through an academic–community partnership. It demonstrates deep engagement (shared design, recruitment, analysis, and dissemination) consistent with CEnR principles. It is not labeled CBPR to avoid implying complete community control, though it embodies the participatory ethos of co-learning and mutual benefit.

### Ethical Approval

The study received ethical approval from the University of Rochester Research Subjects Review Board (STUDY00007858) and the National Ethics Committee for Clinical Trials of Public Research Bodies and the Istituto Superiore di Sanit in Italy (AOO-ISS - 04/07/2023 – 0031228). Participants were informed about the study’s objectives, procedures, potential risks, and benefits. Written informed consent was obtained from participants. For those with limited literacy, the consent form was read aloud. Data confidentiality was strictly upheld, with all identifying information anonymized in data handling and reporting. Written informed consent was obtained from all interview and focus group participants. For the quantitative survey, informed consent was implied upon completion of the questionnaire following provision of a detailed information sheet explaining the study’s purpose, confidentiality safeguards, and voluntary nature of participation.

## Results

The quantitative survey included 150 RMSWs living in Italy, consisted of 20 in-depth interviews, and two focus group discussions with ARMSWs. Participants shared detailed narratives of their pathways into sex work, including experiences in their home countries, during migration, and in Italy. Collectively, the sample highlights a population shaped by precarity, forced mobility, and intersecting stigmas.

### Participant Characteristics

The participants in the qualitative study are primarily African male refugees who are sex workers living in Italy. **Error! Reference source not found.** summarizes their demographic characteristics. Most participants (96.97%) identified as male, with a median age of 30 years. Nearly half (47.33%) identified as Christian, while 57.33% reported being single. In addition, 87.33% were refugees, and 57.33% were of Nigerian origin.

### Quantitative results

#### Socio-economic and Personal Drivers of Engagement in Sex Work

Survey data revealed that sex work was not a supplementary source of income but the primary livelihood strategy for most participants. A substantial majority (79%) reported relying exclusively on sex work as their sole source of income, reflecting the severe lack of alternative employment opportunities available to ARMSWs in Italy. Early initiation into commercial sex was also common. Approximately 17% of participants began engaging in sex work at age 18 or younger, and more than two-thirds (72%) had entered by the age of 25, underscoring how structural exclusion and precarity during adolescence and early adulthood shaped their pathways into sex work. Only a small minority (21%) reported holding any additional form of employment outside of sex work, further highlighting the dependence on transactional sex as a means of survival. The Table 1 presents the summary of these findings.

### Economic motivations for engaging in sex work

#### Theme 1- Economic Drivers and Sexual Risk-Taking

A series of chi-square tests of independence was conducted to evaluate the association between engaging in unprotected sex (defined as sex without protection because of being offered money or another reason) and four different types of economic pressures: money for rent, food, children, and having no other income option. Cramér’s V was used to assess the strength of association for each test. Table 3 presents the summary of findings.

##### Money for Rent

Participants who reported engaging in unprotected sex were significantly more likely to cite the need for money to pay rent as a motivating factor (*χ*^2^ = 5.81, *p* = 0.016). Specifically, 59% of those who engaged in unprotected sex did so due to rent-related financial need, compared to 41% who did not. The strength of this association, as measured by Cramér’s V (0.21), indicates a small to moderate effect size, suggesting that housing instability may contribute to sexual risk-taking behavior.

##### Money for food

A similar pattern was observed for participants who indicated that the need for food influenced their decision. The association between engaging in unprotected sex and needing money for food was statistically significant (*χ*^2^ = 5.77, *p* = 0.016), with 59% of those who engaged in unprotected sex reporting food insecurity, versus 41% of those who did not. Cramér’s V was also 0.21, consistent with a small to moderate association, underscoring the role of basic subsistence needs in shaping sexual health decisions.

##### No other income option

Participants who indicated that they had no other income option were significantly more likely to engage in unprotected sex (*χ*^2^ = 4.377, *p* = 0.036). Of those who engaged in unprotected sex, 54% reported this circumstance, while only 46% did not. The Cramér’s V value of 0.18 indicates a small effect size, suggesting that the lack of alternative income sources contributes to vulnerability in sexual health decision-making.

#### Theme 2: Economic Motivations and HIV Service Awareness

Food insecurity was linked to lower knowledge of HIV services (χ^2^ = 10.19, p = 0.001, Cramér’s V = 0.26). Having another job (χ^2^ = 4.80, p = 0.028, Cramér’s V = 0.18) and willingness to change jobs (χ^2^ = 15.65, p < 0.001, Cramér’s V = 0.32) were also associated with awareness, suggesting occupational precarity influences access to information. HIV self-testing awareness was highest among those willing to change jobs (χ^2^ = 20.32, p < 0.001, Cramér’s V = 0.37).

This theme illustrates how economic precarity, especially the need for basic necessities like food, can impact awareness and knowledge of HIV services. Individuals who entered sex work due to the urgent need for food were significantly less likely to know where to access HIV testing and care. Similarly, those who had supplementary income from other jobs or were unwilling to change their profession (even if pay was the same) also demonstrated lower HIV service awareness. The relatively high Cramér’s V values (ranging from 0.18 to 0.37) underscore moderate associations, especially with self-testing awareness and occupational flexibility. These patterns suggest that those who feel economically trapped in sex work may be more isolated from health information, while those who express a willingness to exit are better informed, perhaps due to broader social networks or resource access (Table 4).

#### Theme 3: Economic Drivers and Use of Traditional Medicine

Reliance on herbal remedies was more common among participants citing food insecurity (χ^2^ = 4.57, p = 0.032, Cramér’s V = 0.17), rent obligations (χ^2^ = 6.83, p = 0.009, Cramér’s V = 0.21), or no other income (χ^2^ = 4.41, p = 0.036, Cramér’s V = 0.17). This cluster emphasizes how financial instability correlates with the use of traditional herbal remedies. Participants who cited food insecurity, lack of income, or rent obligations as their primary reason for entering sex work were significantly more likely to resort to traditional medicine (Table 5). These associations, although moderate, indicate a tendency among economically vulnerable individuals to rely on accessible or culturally familiar healthcare options, potentially due to limited access to formal health systems. This also hints at broader issues of healthcare access and trust among marginalized communities.

#### Theme 3: Economic Hardship, Stigma, and Unmet Needs

Food insecurity was linked to greater hospital stigma (χ^2^ = 4.43, p = 0.035, Cramér’s V = 0.17), while those with no other income were more likely to report unmet healthcare needs (χ^2^ = 13.30, p = 0.001, Cramér’s V = 0.30). These findings highlight how economic vulnerability intersects with experiences of healthcare-related stigma and unmet needs. Individuals who entered sex work due to food insecurity were more likely to face discrimination in hospital settings related to their identity as immigrants, sex workers, or sexual minorities (Table 6). Similarly, those with no alternative income sources reported higher unmet healthcare needs within their communities. This underscores how economic hardship amplifies marginalization within the healthcare system, further compromising health outcomes for already vulnerable populations.

#### Theme 5: Economic Motivation and Sexual Health Behaviors

Economic stressors were associated with protective behaviors, but with mixed outcomes. For example, condom use was higher among those citing food (χ^2^ = 11.34, p < 0.001, Cramér’s V = 0.29), rent (χ^2^ = 5.18, p = 0.022, Cramér’s V = 0.19), or childcare (χ^2^ = 5.90, p = 0.015, Cramér’s V = 0.21) as motivators, as well as those without other income options (χ^2^ = 13.87, p < 0.001, Cramér’s V = 0.32). However, lack of income was also associated with poorer HIV testing outcomes (χ^2^ = 13.77, p = 0.003, Cramér’s V = 0.30). Willingness to adopt HIV self-testing was greater among those motivated by food (χ^2^ = 9.22, p = 0.010, Cramér’s V = 0.25) or lack of other income (χ^2^ = 20.99, p < 0.001, Cramér’s V = 0.37). Sexual health behaviors, particularly condom use, HIV testing, and knowledge of partners, were strongly linked to the economic motivations behind entering sex work. Individuals motivated by food insecurity, childcare, rent, or lack of alternative income showed distinct patterns. For example, those facing financial desperation were more likely to use condoms, perhaps reflecting heightened risk awareness. However, some subgroups also showed lower levels of partner knowledge or reported worse testing outcomes, indicating complex dynamics between financial stress, sexual health practices, and exposure to HIV risk. The consistently moderate-to-strong associations (Cramér’s V up to 0.32) across this theme emphasize the deeply intertwined nature of economic hardship and HIV vulnerability (Table 7)

### Qualitative Results

#### 1. Economic and Personal Reasons for Entering Sex Work

Participants consistently described entering sex work due to economic pressures. In their countries of origin, unemployment, poverty, and responsibility toward family members were frequently reported as primary motivations. One participant explained (P2), “Because Nigeria, you don’t have job… there’s no job. So you have to find something good for yourself… like sex work.” Another noted beginning sex work to support his family (P3): “I decided to become a sex worker because I was too young and could not get a job… I really don’t want my father to suffer. I want to give him the best of life.” For others, sex work was not driven by desire but by the need to meet basic obligations: “Sex work was never fun. I had to do it for my commitments, for my family, my bills” (P4).

Migration further intensified these economic constraints. Participants described being compelled to exchange sex for food, transport, or survival during their journey toward Italy. These experiences were framed as coerced by circumstance rather than voluntary. As one participant recounted (P5), “All of the countries I transited, I did sex work… even though you don’t want to do it, you will do it with force to survive.” Another explained how transactional sex became part of surviving border crossings (P6): “Yes, it was crazy from Nigeria, to Niger, to Algeria, and then Libya… I had to pay twice… till I found favor from one of the Arab guys who liked me.” A third described the violent treatment presented during transit (P7): “They took four, four from the guys… it’s a way of being forced because you have two options… your life or sex.”

Upon arrival in Italy, structural exclusion from employment and persistent financial instability continued to shape engagement in sex work. Participants reported relying on sex work as their only viable source of income to cover housing, food, and daily expenses. One described this transition clearly (P8): “Actually, before when I came to Europe, I was not doing that for money… But later, when I came to Europe here, I noticed that you can make some money from it.” Another emphasized the link to unemployment (P9): “Job is one of the greatest problems here (Italy) and it’s part of why some of us go into sex working just to gain a living.” For others, sex work also financed education or personal survival needs (P10): “Well it wasn’t so interesting because there was never love but I did it just to survive… I did it to pay my studies too.”

Overall, participants’ pathways into sex work were shaped by financial pressures at home, forced survival exchanges during migration, and exclusion from paid employment in Italy.

#### 2. Economic Pressure Shaping Sexual Decision-Making in Sex Work

Participants described how financial need directly influenced sexual decision-making with clients. Several reported engaging in unprotected sex in the past because of financial pressure. One participant reflected on this early period (P1): “Yeah, like…the times I had sexually unprotected sex… I was ignorant and was just interested in the money. I wasn’t interested about my health.”

Economic incentives from clients were a common source of pressure. Participants described clients offering higher payment in exchange of condomless sex. As one explained (P16), “Some clients will be trying to oppress, like trying to pay you more money to have more sex with them.” Others adjusted prices when clients requested condomless sex, linking financial negotiation directly to the health risk involved. One participant described (P19): “Yeah, it’s different money because without using a condom it’s like you are risking your life to someone else… that’s why you need to charge him very well… many people don’t like to use condom.”

At times, financial pressures left participants feeling unable to refuse condomless sex. One participant stated (P20), “If if someone came to you that they want to have sex, without no condom, you have to do… So if you want sex without a condom, I give you my price.” Another emphasized how financial need shapes compromises (P17): “Sometimes people do a lot of things because of money. What you don’t want to do you do it because of money.”

Participants also described clients attempting to force condomless sex under the influence of drugs or alcohol. One described this dynamic (P15): “Some clients often take drugs… some like to make sex with you without protection, trying to force you… while some they get drunk and start misbehaving, also not paying you”

#### 3. Economic Hardship Shaping HIV Testing and Health-Seeking Related to Sex Work

Financial barriers were a central reason participants delayed or avoided HIV testing and care. Private HIV testing was widely described as unaffordable. One participant explained (P7), “Why should I go to the hospital to spend €40 to run a test when even the €40 can last me to feed myself for two days?” Another detailed how lack of money prevented access to testing in both private and some public facilities (P4): “When you go to the private hospital, they charge you for HIV test also if you do sex work… they don’t have the money to carry out the tests, so they become ignorant of it.”

Participants noted that economic insecurity forced trade-offs between health care and immediate needs such as food and shelter. As one explained (P10), “For me I will say it is €20 because this is what I can provide, but for some other persons… €20 out of my salary… it’s too much.”

Economic hardship also pushed some participants toward alternative or traditional remedies when formal healthcare felt inaccessible due to cost. Workers used home-based strategies for managing risk because they could not always afford to attend health services. As one participant described (P17), “Sometimes you may not have money to go to the hospital… so one have to have it at home, for security reasons.”

Participants repeatedly emphasized that free, community-based testing specifically for sex workers would reduce financial barriers. One explained (P6), “If the government or the LGBT community can have a free test center for LGBT sex workers it will help a whole lot of people.”

Across accounts, financial pressure was the primary factor limiting HIV testing and care, shaping health-seeking decisions in ways directly linked to income generated through sex work.

#### 4. Financial Precarity Shaping Power Dynamics with Clients in Sex Work

Participants described how economic vulnerability enhanced clients’ ability to exploit or manipulate them during sex-work encounters. Many reported theft, non-payment, or abrupt changes in agreed terms. As one explained (P3), “Some clients don’t want to pay. Some change the agreement after everything is done. Some try to force you into things you don’t want to do.”

Others described how clients used their financial and social power to intimidate workers, especially when they perceived the sex worker’s economic dependence. One participant recounted (P8), “You are an African, you are a commoner, I can treat you the way I like… I can call the police on you.”

Participants also described being physically assaulted or robbed in situations they accepted due to financial need. One participant explained (P11), “Some will want to have sex with you… they will pay you your money, later they steal from you, some even up to like pouring tear gas on your eyes… there are some bad parts you will not even want to remember.”

Financial desperation limited the ability to refuse coercive or unsafe clients, shaping power dynamics in favor of those who controlled payment. As one participant summarized (P11), “The worst part of the work is based on your clients… Some want more than you can give, and when you refuse, you become the bad person.”

### Integrated Insights

Across both strands, economic precarity emerged as the primary determinant of entry into sex work, sexual risk-taking, and HIV-related health behaviors. Quantitative associations between financial hardship, lower HIV-service awareness, and condomless sex were clarified by qualitative mechanisms showing how economic pressure produces these outcomes: exclusion from formal employment pushed participants into sole reliance on sex work; dependence on client payment weakened their ability to refuse unsafe acts; and cost barriers created direct trade-offs between testing and essential needs such as food and rent. Interview narratives also explained the quantitative association between financial hardship and reliance on traditional remedies, showing that high testing costs led participants to delay care and substitute home-based treatments. While survey data indicated generally strong protective intentions, qualitative accounts demonstrated that financial dependence and client coercion frequently overrode these intentions in practice, producing the observed divergence between intention and behavior. Overall, the integrated findings show that financial instability drives entry into sex work, creates dependency structures that weaken condom negotiation, and produces cost-driven delays in testing and care, generating a continuous cycle of economic and health vulnerability for ARMSWs in Italy.

## Discussion

This study examined how economic precarity shapes HIV vulnerability among ARMSWs in Italy. The study addressed an important gap in the literature. Although recent epidemiological evidence has shown disproportionate HIV and STI burden in this population, there has been limited understanding of how financial pressures, social exclusion, and labor inequality produce heightened exposure to sexual risk (Abu-Ba’are et al., 2025; Nichols et al., 2025). Prior research often framed HIV prevention as a matter of behavior change. However, by integrating quantitative associations with qualitative narratives, this study demonstrates that HIV risk can emerge from severe economic disadvantage that constrains agency, access to prevention services, and negotiation of safer sexual practices. Mixed methods were essential to reveal the mechanisms through which poverty, hunger, and housing instability directly shape sexual and health-seeking decisions among men whose only available survival strategy is sex work (Benner, 2022; Platt et al., 2013). Our results reveal that sex work is overwhelmingly driven by survival imperatives rather than choice or preference, with 79% of participants reporting it as their sole source of income. Our analysis of qualitative and quantitative data converges to show that food insecurity, housing instability, family responsibilities, and lack of alternative employment opportunities are the central motivations for engaging in commercial sex. Importantly, these drivers are not confined to life in Italy but emerge across multiple life stages, including early poverty in participants’ home countries, survival sex during migration journeys, and continued precarity after settlement in Europe.

Thus, our findings revealed that financial hardship consistently emerged as the primary determinant of sex work participation. The quantitative data showed that most participants depended entirely on sex work for basic income, and that food insecurity and rent obligations were significantly associated with engagement in condomless sex. The qualitative findings reinforced these patterns by revealing that men frequently entered sex work after exhausting all other livelihood options, often following prolonged unemployment, homelessness, or migration-related financial crisis. These converging results expand structural vulnerability theory by demonstrating how the lack of legal work authorization and systemic labor exclusion push racialized refugees into high-risk informal economies where HIV risk is unavoidable (Bourgois et al., 2017; Deering et al., 2014). This finding complements growing evidence that African migrant sex workers in Italy face some of the most extreme socioeconomic disadvantages of any subpopulation living with or vulnerable to HIV (Abu-Ba’are et al., 2025; Nichols et al., 2025). The data also expand on research demonstrating how male and LGBTQ+ migrants face unique vulnerabilities in the European sex economy due to intersecting axes of racism, xenophobia, homophobia, and precarious legal status (Dodsworth, 2015; Mai, 2013). The fact that nearly one in five participants began sex work before the age of 18 underscores how poverty and lack of opportunities foster early entry into sexual labor, a finding consistent with evidence linking child and youth poverty to commercial sexual exploitation (Dodsworth, 2015).

One of our strongest integrated findings concerns economic coercion in sexual transactions. Quantitatively, financial need was associated with condomless sex, specifically when clients were offered more money. In our qualitative findings, participants described coercion in which clients refused to pay if a condom was requested. These interactions reflect exploitative power dynamics in which the client holds economic leverage over the worker. This dynamic is well supported in international evidence showing that condom negotiation is undermined when individuals rely on sex work income for survival (Benner, 2022; Platt et al., 2013). This study provides additional granularity by demonstrating that risk-taking is not a result of a lack of knowledge or personal disregard for risk but a survival calculation in response to structural instability. These findings parallel recent work identifying a Syndemic relationship between stress, substance use, exploitation, and high-risk sexual practices among ARMSWs in Italy, where multiple co-occurring vulnerabilities amplify HIV exposure (Nichols et al., 2025).

Economic hardship also played a critical role in shaping HIV prevention access. Our quantitative results showed that those experiencing severe financial distress had lower HIV service awareness and greater reliance on traditional medicine. Qualitative data revealed that clinic visits were often avoided because the costs associated with transportation, lost work time, and stigma outweighed perceived benefits. Many participants indicated that urgent priorities such as obtaining food or paying for temporary shelter made health-seeking behaviors feel like economically irrational decisions. These findings align with known patterns across Europe, where migrant populations are disproportionately likely to receive late HIV diagnosis due to cost barriers, distrust, and fear of discrimination (Logie et al., 2022). Structural discrimination in health systems further discourages follow-up. Evidence from related populations shows that stigma, service refusal, and policing of bodies labeled deviant reinforce avoidance of prevention and care services, particularly when combined with economic marginalization (Abu-Ba’are et al., 2025; Shamrock et al., 2024).

The narratives around survival sex during migration highlight the extent of sexual exploitation faced by ARMSWs. Many participants reported forced sex by smugglers in transit countries, often framed as a choice between life and sexual servitude. This aligns with reports from MØdecins Sans FrontiŁres (2017) (MSF Report, n.d.) and the United Nations High Commissioner for Refugees (On This Journey, No One Cares If You Live or Die, 2020), which documents widespread sexual violence against male migrants on the Central Mediterranean route. Such experiences illustrate how sexual labor is not simply an economic adaptation but also imposed under coercive and violent circumstances, reinforcing the conceptualization of sex work in this population as a continuum of exploitation, survival, and agency.

Although racism did not appear as a primary theme, qualitative narratives indicated that racism often manifests as a form of economic exploitation (Platt et al., 2013). Participants reported experiences where clients used racialized assumptions about African men’s legal vulnerability to withhold payment or threaten reporting to authorities if safety boundaries were asserted. These dynamics intensify dependency on client money and thus elevate the risk of HIV exposure. Prior research similarly shows that anti-Black racism deepens economic inequality and restricts social protections, making marginalized populations more susceptible to coercive sexual labor and reduced healthcare access (Logie et al., 2011; Shamrock et al., 2023). The data here suggest that racism does not merely coexist with poverty but reinforces it, producing compounding structural harms.

The integration of both data strands clarifies that HIV exposure in this population is not a matter of individual irresponsibility but rather the predictable result of structural inequities. Quantitative data established clear statistical relationships between economic factors and risk, while qualitative accounts revealed how these structural conditions shape daily survival decisions. Without this integration, analyses could incorrectly attribute risk behavior to personal choice rather than constrained agency. The mixed methods approach thus offered a more ethically and scientifically accurate explanation of how and why HIV vulnerability persists among ARMSWs in Italy.

These findings align with evidence that financial empowerment and legal labor inclusion serve as critical protective factors for sex workers by enhancing autonomy and reducing exploitation (Deering et al., 2014; Platt et al., 2013). Innovations such as machine-learning-enhanced outreach show promise in identifying needs for individuals who face structural barriers to service access, and in increasing HIV prevention uptake in stigmatized migrant populations (Abu-Ba’Are et al., 2025b). Multilevel interventions implemented in sub-saharan African contexts demonstrate that economic support combined with stigma reduction can improve uptake of testing and PrEP among sexual minority men (Abu-Ba’are et al., 2023, 2024; Shamrock et al., 2024). Although the context differs, the underlying principle is directly relevant. HIV prevention efforts will remain insufficient in Italy so long as the economic drivers that force men into survival-based sex work remain unaddressed.

Collectively, the results have strong policy implications. First, economic deprivation must be recognized as a primary driver of HIV vulnerability. Interventions should center on social protection measures such as food and housing stability, wage subsidization, and guaranteed income programs. Second, refugee labor rights reforms are necessary to expand access to lawful employment and reduce reliance on informal survival economies. Third, HIV prevention services should be redesigned to reduce financial risk, including free, community-based, mobile, and multilingual healthcare delivery. Recent European insights show that effective health equity interventions depend on collaborative governance that empowers communities most affected to shape decisions and program design (Barracchia et al., 2025). Policymakers can apply these frameworks to ensure that refugee sex workers are not excluded from emerging regional health equity agendas. Fifth, HIV surveillance in Italy must move beyond the blanket category of “foreigners” to provide disaggregated data on migrants by sex, gender identity, sexuality, region, and migration background, as accurate classification is critical for identifying distinct subpopulations with specific needs and for designing effective, targeted HIV prevention and testing interventions.

This study makes a meaningful contribution to mixed-methods HIV research and theory. It shows that economic disadvantage is not simply a co-factor but a fundamental cause of HIV risk among marginalized populations. It also highlights the value of research designs that elevate lived experience to explain why statistically observed patterns occur. Future studies should use longitudinal mixed methods to monitor how changes in legal protections, income stability, or social support influence HIV outcomes. Additionally, intervention trials should evaluate whether policies addressing financial precarity produce greater reductions in HIV incidence than behavior-only strategies.

## Limitations

This study has several limitations. First, as a mixed-methods study focused on Northern Italy, the findings may not capture the full diversity of experiences among ARMSWs in other regions or countries. The use of snowball and venue-based sampling may have biased the sample toward individuals already connected to community networks, potentially underrepresenting the most isolated workers; therefore, the results should not be generalized beyond this study population. Second, although the effect sizes observed in the quantitative analyses were small to moderate and statistically significant, the cross-sectional design precludes causal inference. Future quasi-experimental or longitudinal designs, including regression-based approaches, are needed to better assess directionality. Third, reliance on self-reported data introduces the possibility of recall and social desirability bias, particularly regarding sexual practices and experiences of exploitation. Nevertheless, triangulation of quantitative and qualitative findings and strong community partnerships enhance the credibility and contextual validity of our conclusions.

## Conclusion

In conclusion, this study provides compelling evidence that economic insecurity is the central determinant driving sex work involvement and HIV vulnerability among ARMSWs in Italy. Financial hardship shapes entry into sex work, undermines the ability to negotiate safer sex, and reduces uptake of HIV prevention and treatment services. The findings show that HIV risk is structurally produced rather than individually chosen. Public health success will require structural reforms that improve economic conditions, disrupt exploitative power imbalances, and prioritize accessible prevention services. Without addressing the structural forces that make sex work the only viable option for survival, HIV prevention for this population will continue to fall short.

## Figures and Tables

**Table 1.** Sociodemographic Characteristics of Participants (N = 150)

Demographic	n (%) or Mean ± SD
Age (years)	30.6 ± 5.9
Education Level	Senior high school/vocational, 74 (49.3)
Gender Identity	Man, 145 (96.7) Transgender, 3 (2.0) Non-binary, 2 (1.3)
Marital Status	Single, 86 (57.3) Married, 53 (35.3) Divorced, 10 (6.7) Widowed, 1 (0.7)
Number of Children	None, 80 (53.3) One, 25 (16.7) Two, 26 (17.3) Three, 13 (8.7) Four, 6 (4)
Religious Affiliation	Christianity, 71 (47.3) Islam, 37 (24.7) African Traditional Religion, 16 (10.7) No religion, 25 (16.7) Other 1 (0.7)
Immigration Status	Refugee, 131 (87.3)
Country of Origin	Nigeria, 86 (57.3) Ghana, 16 (10.7) Cameroon, 6 (4.0)

*Note*. Values are presented as n (%) unless otherwise indicated.

**Table 1.** Socio-Economic Drivers of Sex Work Among ARMSWs (N = 150)

Variable	n (%)
Sex work as a sole source of income	118 (79.0)
Additional employment besides sex work	32 (21.0)
Began sex work at age ≤ 18	25 (16.7)
Began sex work at age ≤ 25	108 (72.0)

**Table 3.** Associations Between Economic Drivers and Engagement in Unprotected Sex (χ^2^ and Cramér’s V)

Have you ever had sex without using protection (like condoms) because you were offered money or for some other reason?					
	Frequency (%)	Yes (n=78) Obs (Exp)	No (n=72) Obs (Exp)	*χ*^2^-P-value	Cramér’s V (0–1)
**money for rent**				5.81– **0.016**	0.21
**yes**	88 (59%)	38 (45.76)	50 (42.24)		
**no**	62 (41%)	40 (32.24)	22 (29.76)		
**money for food**				5.770– **0.016**	0.21
**yes**	89 (59%)	40 (46.28)	35 (42.72)		
**no**	61 (41%)	24 (31.72)	37 (29.28)		
**money for children**				0.793– 0.373	0.09
**yes**	65 (43%)	37 (33.80)	28 (31.20)		
**no**	85 (57%)	41 (44.20)	44 (40.80)		
**no other income option**				4.377– **0.036**	0.18
**yes**	81 (54%)	49 (42.12)	32 (38.88)		
**no**	69 (46%)	29 (35.88)	40 (33.12)		
**Do you have any other job you do apart from sex work?**				0.729– 0.393	0.09
**yes**	32 (21%)	14 (16.64)	18 (15.36)		
**no**	118 (79%)	64 (61.36)	54 (56.64)		

**Table 4.** Associations Between Economic Drivers and HIV Service Awareness (χ^2^ and Cramér’s V)

Hypothesis	*χ*^2^- P-value	Cramér’s V
**Entering sex work due to need for food ↔ HIV service awareness (Obs=24, Exp=33.82)**	10.187 – 0.001	0.26
**Having other jobs ↔ HIV service awareness (Obs=14, Exp=19.84)**	4.80 – 0.028	0.18
**Willingness to change jobs ↔ HIV service awareness (Obs=43, Exp=30.78)**	15.65 – <0.001	0.32
**Entering sex work due to food insecurity ↔ HIV service awareness (Obs=28, Exp=37.82)**	10.19 – 0.001	0.26
**HIV self-testing awareness ↔ Willingness to change jobs (Obs=59, Exp=44.82)**	20.32 – <0.001	0.37

**Table 5.** Associations Between Economic Drivers and Use of Traditional or Herbal Medicine (χ^2^ and Cramér’s V)

Hypothesis	*χ*^2^- P-value	Cramér’s V
**Need for food ↔ Use of traditional medicine (Obs=28, Exp=21.95)**	4.57 – 0.032	0.17
**Need for rent ↔ Use of traditional medicine (Obs=29, Exp=21.71)**	6.83 – 0.009	0.21
**No other income ↔ Use of traditional medicine (Obs=26, Exp=19.98)**	4.41 – 0.036	0.17

**Table 6.** Associations Between Economic Drivers and Healthcare Barriers, Including Hospital Stigma and Unmet Healthcare Needs (χ^2^ and Cramér’s V)

Hypothesis	*χ*^2^- P-value	Cramér’s V
**Need for food ↔ Hospital stigma/discrimination (Obs=38, Exp=31.45)**	4.43 – 0.035	0.17
**No other income ↔ Unmet healthcare needs (Obs=31, Exp=22.14)**	13.30 – 0.001	0.30

**Table 7.** Extended Associations Between Economic Drivers and Sexual Health Indicators (χ^2^ and Cramér’s V)

Hypothesis	*χ*^2^- P-value	Cramér’s V
**Willingness to change jobs ↔ Condom use (Obs=21, Exp=13.14)**	10.63 – 0.001	0.28
**Need for food ↔ Condom use (Obs=75, Exp=67.03)**	11.34 – <0.001	0.29
**No other income ↔ Condom use (Obs=71, Exp=62.13)**	13.87 – <0.001	0.32
**Need for children ↔ Condom use (Obs=55, Exp=49.05)**	5.90 – 0.015	0.21
**Need for rent ↔ Condom use (Obs=20, Exp=14.41)**	5.18 – 0.022	0.19
**Need for rent ↔ Knowing partner history (Obs=21, Exp=29.92)**	8.69 – 0.003	0.24
**Need for children ↔ Knowing partner history (Obs=32, Exp=22.1)**	10.69 – 0.001	0.27
**No other income ↔ HIV test result (Obs=25, Exp=18.86)**	13.77 – 0.003	0.30
**Need for food ↔ HIV self-testing willingness (Obs=42, Exp=36.19)**	9.22 – 0.010	0.25
**No other income ↔ HIV self-testing willingness (Obs=46, Exp=32.94)**	20.99 – <0.001	0.37

**Table 8.** Joint Display Integrating Quantitative Findings with Qualitative Themes

Domain	Quantitative Evidence	Qualitative Evidence	Integration
**1. Economic and Personal Reasons for Entering Sex Work**	79% rely solely on sex work; 72% entered by age ≤25	Poverty, unemployment, family responsibilities; survival sex during migration; no jobs in Italy	**Strong convergence:** Sex work is a survival income strategy shaped by structural exclusion at origin, during migration, and in Italy.
**2. Economic Pressure Shaping Sexual Decision-Making**	Rent, food, and no income significantly associated with unprotected sex (p ≤ .036)	Extra payment for condomless sex; coercive negotiation; inability to refuse due to financial need; intoxicated/violent clients pushing unsafe sex	**Convergence with mechanism:** Economic incentives and dependency explain statistical patterns. **Divergence clarified:** Quantitative “protective” patterns reflect intent; qualitative shows this intent is often undermined by coercion and dependence.
**3. Economic Hardship and HIV Testing/Health-Seeking**	€40 test cost = key barrier; food insecurity linked to lower service awareness (p=.001), higher hospital stigma (p=.035), and unmet needs (p=.001); economic drivers predict traditionalmedicine use (p ≤ .036)	Testing avoided due to cost; prioritizing food/rent over care; home-based remedies used when hospitals unaffordable; desire for free sex-work-specific testing	**Strong convergence:** Financial constraints directly limit testing, shape care decisions, and push participants to alternative remedies; qualitative narratives explain *why* these associations occur.
**4. Financial Precarity Shaping Power Dynamics with Clients**	Economic dependence linked to unprotected sex and poorer HIV outcomes	Non-payment, theft, intimidation; being treated as disposable; inability to refuse unsafe or abusive clients	**Convergence:** Financial vulnerability increases exposure to coercion and exploitation, reinforcing both sexual and economic risk cycles.

## Data Availability

Due to the extreme vulnerability and legal precarity of the study population, the datasets generated and/or analysed during the current study are not publicly available. De-identified data may be available from the corresponding author on reasonable request and subject to additional ethical and data protection review.
